# The effect of aqueous extract of *Embelia ribes* Burm on serum homocysteine, lipids and oxidative enzymes in methionine induced hyperhomocysteinemia

**DOI:** 10.4103/0253-7613.43161

**Published:** 2008-08

**Authors:** Uma Bhandari, M. Nazam Ansari, F. Islam, C.D. Tripathi

**Affiliations:** Department of Pharmacology, Faculty of Pharmacy, Hamdard University, New Delhi, India; 1 Department of Toxicology, Faculty of Science, Hamdard University, New Delhi, India; 2 Department of Pharmacology, Vardhman Mahavir Medical College, Safdarjung Hospital, New Delhi, India

**Keywords:** *Embelia ribes*, homocysteine, hyperlipidemia, methionine, oxidative stress

## Abstract

**Objective:**

The present study was designed to evaluate the effect of the aqueous extract of *Embelia ribes* Burm fruits on methionine-induced hyperhomocysteinemia, hyperlipidemia and oxidative stress in albino rats.

**Materials and Methods:**

Adult male Wistar albino rats were fed with the aqueous extract of *Embelia ribes* (100 and 200 mg/kg, p.o.) for 30 days. Hyperhomocysteinemia was induced by methionine treatment (1 g/kg, p.o.) for 30 days and folic acid (100 mg/kg, p.o.) was used as a standard drug. The animals were evaluated for various biochemical parameters in serum and brain homogenates, followed by histopathological studies at the end of the study.

**Results:**

Administration of methionine (1 g/kg, p.o.) for 30 days to vehicle control rats produced significant increase (*P* < 0.01) in homocysteine, lactate dehydrogenase (LDH), total cholesterol, triglycerides, low density lipoprotein (LDL-C), very low density lipoprotein (VLDL-C) levels in serum and lipid peroxides (LPO) levels in brain homogenates, with reduction in high density lipoprotein (HDL-C) levels in serum, and glutathione (GSH) content in brain homogenates, as compared to vehicle control rats. Administration of the aqueous extract of *Embelia ribes* (100 and 200 mg/kg, p.o.) for 30 days, to hyperhomocysteinemic rats, significantly (*P* < 0.01) decreased the levels of homocysteine, LDH, total cholesterol, triglycerides, LDL-C and VLDL-C and increased the HDL-C levels in serum. In addition, a significant (*P* < 0.01) decrease in LPO levels with increase in GSH content was observed in hyperhomocysteinemic rats treated with the aqueous extract of *Embelia ribes*. The results were comparable to those obtained with folic acid, a standard antihyperhomocysteinemic drug.

**Conclusion:**

The present results provide clear evidence that the aqueous extract of *Embelia ribes* treatment enhances the antioxidant defense against methionine-induced hyperhomocysteinemia, hyperlipidemia and oxidative stress in brain.

## Introduction

Hyperhomocysteinemia is a risk factor for coronary atherosclerotic vascular disease, stroke, and venous thrombosis. It has been associated with Alzheimer's disease and vascular dementia.[1–4] Although its pathophysiological mechanisms are complex and not fully understood, much evidence suggests that hyperhomocysteinemia induces vascular and brain damage because of the highly reactive thiol group in homocysteine, which is readily oxidized, leading to the formation of homocysteine, homocysteine mixed disulfides and homocysteine thiolactone. During these oxidative processes, several reactive species are generated.[2] The methionine cycle is responsible for the formation of homocysteine in the body.

Homocysteine is toxic sulfur containing intermediate amino acid, which results from intracellular conversion of methionine to cysteine, which can induce neuronal dysfunction and cell death. Homocysteine has now been implicated in increased oxidative stress, DNA damage, triggering of apoptosis and excitotoxicity, all important mechanisms in neurodegeneration.[5] The brain may be particularly vulnerable to high levels of homocysteine in the blood because it lacks two major metabolic pathway for its elimination - betain remethylation and transsulfuration.[6] Homocysteine is proposed to cause oxidative stress related neurotoxicity[7] and natural antioxidants play a protective role in hyperhomocysteinemia;[8] however, there is a paucity of experimental as well as clinical studies which indicates the protective role of medicinal plants in hyperhomocysteinemia.

*Embelia ribes* Burm, commonly known as ‘Vidanga’, is a large woody climbing shrub that belongs to the family, *Myrsinaceae*, which is widely distributed in India, Sri Lanka, Malaysia and South China.[9] It is highly valued in Ayurveda as a powerful anthelmintic.[10] In a preliminary study, Tripathi[11] has demonstrated the blood glucose lowering activity of the decoction of the *Embelia ribes* fruits in glucose-fed albino rabbits. Bhandari *et al.*[12,13] have reported the diabetic dyslipidemic and antioxidant activity of *Embelia ribes* Burm in streptozotocin-induced diabetic rats, using gliclazide as a positive control drug. Recently, Bhandari *et al.*[14] have reported the cardioprotective activity of the aqueous extract of *Embelia ribes,* in isoproterenol induced myocardial infarction in albino rats.

However, till date, antihyperhomocysteinemic activity has not been carried out on the aqueous extract of *Embelia ribes*. Therefore, the present study was undertaken to determine the antihyperhomocysteinemic and antihyperlipidemic activity of the aqueous extract of *Embelia ribes* in albino rats.

## Materials and Methods

### Drugs and chemicals

Methionine and folic acid were obtained from Central Drug House, Bombay. All the other chemicals that were used were of analytical grade. Double distilled water was used for all biochemical assays.

### Preparation of aqueous extract of Embelia ribes Burm

Dried fruits of *Embelia ribes* Burm were purchased from the local market, New Delhi, India, in October 2006 and the botanical authentification was carried out by the Department of Botany, Faculty of Science, Hamdard University, New Delhi, India (voucher specimen no. UB 2). The dried and coarsely powdered drug (100 g) was packed in a soxhlet apparatus and was subjected to extraction with water for 72 hours. The filtrate was evaporated under a vacuum drier (Narang Scientific Works Pvt. Ltd., New Delhi, India) and the brown mass residue that was obtained was stored at 4°C, for further use. The average yield of the aqueous extract of *Embelia ribes* was approximately 5.26%. For experimental study, the weighed amount of aqueous extract of *Embelia ribes* (100 and 200 mg/kg) was dissolved in 1% Tween 80 in distilled water.

### Standardization of extract

Preliminary phytochemical screening of the aqueous extract of dried fruits of *Embelia ribes* was carried out for the detection of phytoconstituents, using standard chemical tests. Alkaloids, carbohydrates, phenolic compounds, flavonoids, proteins and saponins were detected in the extract. HPTLC fingerprints of the aqueous extract was established using CAMAG HPTLC (WinCAT software, version 2.2) and benzene: ethyl acetate (6:4) as the solvent system, which showed the presence of seven spots (R_f_ values: 0.32, 0.34, 0.42, 0.45, 0.52, 0.65 and 0.78) at 520 nm wavelength.

### Animals

Male adult Wistar albino rats, weighing 200-250 g, procured from the Central Animal House Facility, Hamdard University, New Delhi, were used. The Institutional Animal Ethics Committee (IAEC) approved the present research. Animals were acclimatized under standard laboratory conditions at 25° ± 2°C, 50 ± 15% RH and normal photoperiod (12 h light : dark cycle) for seven days. The animals were fed with commercial rat pellet diet and water *ad libitum*.

### Methionine-induced hyperhomocysteinemia in rats

The animals were randomly divided into seven groups of ten animals each and treated as follows: Group I served as vehicle control and received only 1% tween 80 in distilled water (2 ml/kg, p.o.) for 30 days. Group II served as pathogenic control and received methionine (1 g/kg, p.o.)[15] for 30 days. Groups III and IV were administered aqueous extract of *Embelia ribes* at doses of 100 and 200 mg/kg respectively and co-treated with methionine (1 g/kg, p.o.) for 30 days. Group V served as positive control and received folic acid (100 mg/kg, p.o.)[16] and co-treated with methionine (1 g/kg, p.o.) for 30 days. Groups VI and VII were administered aqueous extract of *Embelia ribes* alone at doses of 100 and 200 mg/kg respectively, for 30 days. The blood samples were collected by the retro-orbital plexus using micro-capillary technique from all the groups of rats that were made to fast overnight. Serum was separated for biochemical estimations.

In serum, homocysteine levels were estimated using the Fluorescence Polarization Immunoassay (FPIA) method described by Primus *et al.* (1988). UV spectrophotometric method of analysis was used for the estimation of LDH (Lum and Gambino, 1974), total cholesterol (Demacher and Hijamaus, 1980), triglycerides (Foster and Dunn, 1973) and HDL-C (Burstein *et al.*, 1970). Commercial diagnostic kits from SPAN Diagnostics, Udhna, Surat, India were used for cholesterol and triglycerides estimation. Lactate dehydrogenase (LDH) catalyses the oxidation of lactate to pyruvate, accompanied by a simultaneous reduction of NAD to NADH. Lactate dehydrogenase (LDH) activity in serum is proportional to the increase in absorbance, due to the reduction of NAD. Lactate dehydrogenase (LDH) and HDL-C levels were estimated using commercial diagnostic kits from Reckon Diagnostics Pvt. Ltd. Baroda, India. LDL-C and VLDL-C levels were calculated from the formula of Friedward *et al.* (1972) as given below:

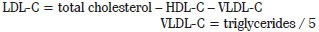



The animals were sacrificed by cervical dislocation and brain tissues were dissected for biochemical estimation and for histopathological studies. Homogenate (10%) of whole brain tissue in ice cold KCl (0.15 M) was used for the assay of the malondialdehyde, according to the method of Ohkawa *et al.* (1979). In phosphate buffer (0.1 M, pH 7.0), it was used for the assay of glutathione (GSH) content (Sedlak and Lindsay, 1968). Lipid peroxides (LPO) was measured by estimating thiobarbituric acid reactive substances (TBARS) i.e. malondialdehyde (MDA), and glutathione assay was based on the reaction with DTNB i.e. DTNB (5,5'-dithiobis- (2-nitrobenzoic acid)) is reduced by -SH group to form one mole of 2-nitro-5-mercaptobenzoic acid (yellow color).

### Histopathological studies

At the end of the experiment, whole brain tissues from all the groups were subjected to histopathological studies. The tissues were fixed in formalin (10%), routinely processed and embedded in paraffin wax. Paraffin sections (5µm thick) were cut on glass slides and stained with hematoxylin and eosin (H & E), after dewaxing, and examined under a light microscope.

### Statistical analysis

The results are expressed as mean±SEM. Statistical differences between means were determined by one-way analysis of variance (anova), followed by Dunnett t-test. Values of *P* < 0.01 were considered significant.

## Results

Preliminary phytochemical screening of the aqueous extract of dried fruits of *Embelia ribes* was carried out and alkaloids, carbohydrates, phenolic compounds, flavonoids, proteins and saponins were detected in the extract. HPTLC fingerprints of aqueous extract showed the presence of seven spots (R_f_ values: 0.32, 0.34, 0.42, 0.45, 0.52, 0.65 and 0.78) at 520 nm wavelength.

Significant (*P* < 0.01) increase in the serum homocysteine and lactate dehydrogenase (LDH) levels were observed in pathogenic control (i.e. group II) rats, as compared to vehicle control rats (group I). Aqueous extract of *Embelia ribes* (group III and IV) significantly (*P* < 0.01) lowered the methionine-induced elevations of serum homocysteine and LDH levels, as compared to pathogenic control rats (group II). The results were comparable to those of folic acid [Table 1].

**Table 1:** Effect of aqueous extract of *Embelia ribes* on serum homocysteine and LDH levels in methionine-induced hyperhomocysteinemic rats

*Group*	*Homocysteine (µg/mL)*	*LDH (IU/L)*
Vehicle control	8.52 ± 0.19	28.30 ± 0.81
Methionine treated	22.65 ± 0.34^a^	59.36 ± 0.69^a^
Aqueous *Embelia ribes* extract (100 mg/kg) + Methionine	16.08 ± 0.03^b^	42.40 ± 0.42^b^
Aqueous *Embelia ribes* extract (200 mg/kg) + Methionine	16.08 ± 0.05^b^	39.03 ± 0.44^b^
Folic Acid (100 mg/kg) + Methionine	14.72 ± 0.03^b^	34.50 ± 0.36^b^
Aqueous *Embelia ribes* extract (100 mg/kg)	9.07 ± 1.00^c^	29.84 ± 0.56^c^
Aqueous *Embelia ribes* extract (200 mg/kg)	9.07 ± 0.10^c^	30.59 ± 1.10^c^

Values are mean ± SEM (n=10);

a *P*<0.01

c *P*>0.05 as compared to vehicle control group

b *P*<0.01 as compared to pathogenic control group

Serum total cholesterol, triglycerides, LDL-C, VLDL-C levels were significantly (*P* < 0.01) increased along with a significant (*P* < 0.01) decrease in HDL-C levels in pathogenic control (i.e. group II) rats, as compared to vehicle control (i.e. group I) rats. With repeated administration of aqueous extract of *Embelia ribes* (group III and IV) and folic acid (group V) treatment, the above mentioned parameters were regained significantly (*P* < 0.01), as compared to pathogenic control (i.e. group II) rats [Tables 2 and 3].

**Table 2:** Effect of aqueous extract of *Embelia ribes* on serum total cholesterol, triglycerides and HDL-C levels in methionine-induced hyperhomocysteinemic rats

*Groups*	*Total Cholesterol (mg/dl)*	*Triglycerides (mg/dl)*	*HDL-C (mg/dl)*
Vehicle control	100.60 ± 0.87	87.15 ± 1.31	39.58 ± 0.57
Methionine treated	194.21 ± 1.65^a^	179.52 ± 2.15^a^	14.23 ± 0.44^a^
Aqueous *Embelia ribes* extract (100 mg/kg) + Methionine	167.66 ± 1.20^b^	142.42 ± 1.52^b^	21.16 ± 0.40^b^
Aqueous *Embelia ribes* extract (200 mg/kg) + Methionine	157.56 ± 1.79^b^	130.63 ± 2.08^b^	23.97 ± 0.51^b^
Folic Acid (100 mg/kg) + Methionine	147.57 ± 1.51^b^	119.23 ± 1.72^b^	30.46 ± 1.10^b^
Aqueous *Embelia ribes* extract (100 mg/kg)	99.57 ± 0.60^c^	86.96 ± 0.68^c^	39.74 ± 0.19^c^
Aqueous *Embelia ribes* extract (200 mg/kg)	97.87 ± 1.20^c^	85.02 ± 1.37^c^	39.15 ± 0.37^c^

Values are mean ± SEM (n=10);

a *P*<0.01

c *P*>0.05 as compared to vehicle control group

b *P*<0.01 as compared to pathogenic control group

**Table 3:** Effect of aqueous extract of *Embelia ribes* on serum LDL-C, VLDL-C levels and atherosclerotic index in methionine-induced hyperhomocysteinemic rats

*Groups*	*LDL-C (mg/dl)*	*VLDL-C (mg/dl)*	*Atherosclerotic Index (cholesterol/HDL-C)*	*Atherosclerotic Index (LDL-C/HDL-C)*
Vehicle control	43.59±0.56	17.43±0.26	2.54±0.02	1.10±0.02
Methionine treated	144.08±1.71^a^	35.90±0.43^a^	13.69±0.41^a^	10.16±0.33^a^
Aqueous *Embelia ribes* extract (100 mg/kg) + Methionine	118.01±0.68^b^	28.48±0.30^b^	7.93±0.11^b^	5.58±0.10^b^
Aqueous *Embelia ribes* extract (200 mg/kg) + Methionine	107.46±0.87^b^	26.12±0.42^b^	6.58±0.07^b^	4.49±0.06^b^
Folic Acid (100 mg/kg) + Methionine	93.46±0.61^b^	23.84±0.34^b^	4.89±0.12^b^	3.10±0.10^b^
Aqueous *Embelia ribes* extract (100 mg/kg)	42.45±0.28^c^	17.39±0.14^c^	2.50±0.01^c^	1.07±0.01^c^
Aqueous *Embelia ribes* extract (200 mg/kg)	41.71±0.56^c^	17.01±0.27^c^	2.50±0.01^c^	1.06±0.01^c^

Values are mean±SEM (n=10);

a *P*<0.01

c *P*>0.05 as compared to vehicle control group

b *P*<0.01 as compared to pathogenic control group

Methionine treatment significantly (*P* < 0.01) increased the LPO levels and decreased the GSH levels in brain homogenates in pathogenic control (i.e. group II) rats, as compared to vehicle control (i.e. group I) rats. Aqueous extract of *Embelia ribes* (group III and IV) and folic acid (group V) treatment in methionine-induced hyperhomocysteinemic rats significantly (*P* < 0.01) decreased the LPO levels and increased the GSH levels in brain homogenates, as compared to pathogenic control (i.e. group II) rats [Table 4].

**Table 4:** Effect of aqueous extract of *Embelia ribes* on LPO and GSH levels in brain in methionine-induced hyperhomocysteinemic rats

*Groups*	*LPO (nmol MDA/mg protein)*	*GSH (µmole of phosphorous liberated/min/mg protein)*
Vehicle control	1.75±0.01	13.63±0.33
Methionine treated	6.87±0.12^a^	8.93±0.47^a^
Aqueous *Embelia ribes* extract (100 mg/kg) + Methionine	4.40±0.09^b^	10.70±0.31^b^
Aqueous *Embelia ribes* extract (200 mg/kg) + Methionine	3.71±0.06^b^	11.02±0.17^b^
Folic Acid (100 mg/kg) + Methionine	3.21±0.04^b^	12.84±0.14^b^
Aqueous *Embelia ribes* extract (100 mg/kg)	1.78±0.04^c^	14.52±0.20^c^
Aqueous *Embelia ribes* extract (200 mg/kg)	1.86±0.03^c^	14.61±0.54^c^

Values are mean±SEM (n=10);

a *P*<0.01

c *P*>0.05 as compared to vehicle control group

b *P*<0.01 as compared to pathogenic control group

**Figure 1:**
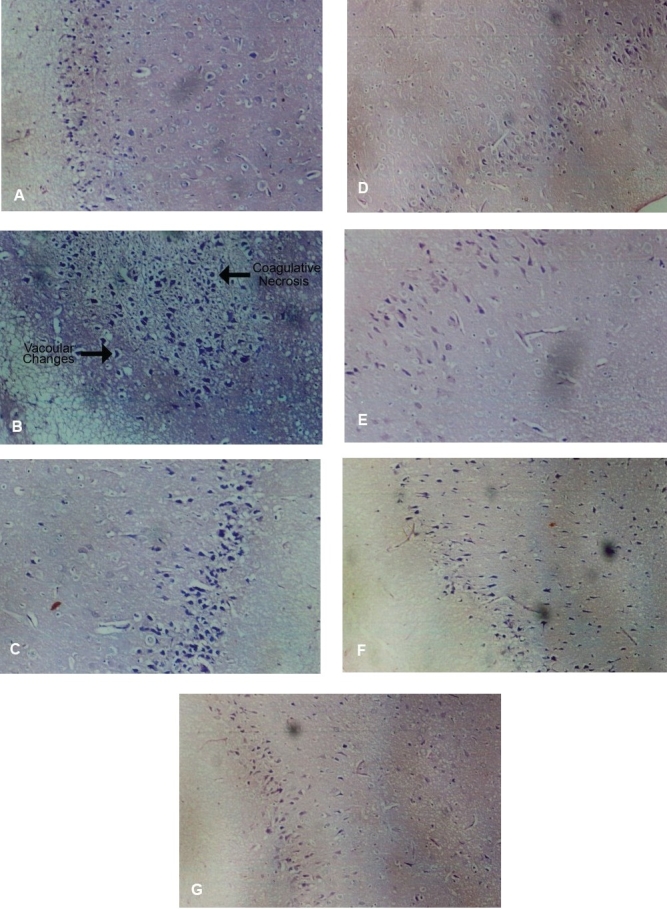
Histology of brain showing normal fibrillary background and neuronal cell morphology (A), methionine-induced coagulative necrosis and vacuolar changes (B), photomicrograph showing normal fibrillary background with absence of degenerative changes or necrosis in Embelia ribes treated rats photomicrograph showing normal fibrillary background with absence of degenerative changes or necrosis in Embelia ribes treated rats (C and D) folic acid treated group showing normal fibrillary background without any pathological symptoms (E), aqueous Embelia ribes extract per se groups showing normal fibrillary background and neuronal cell morphology (F and G) (H&E, ×400)

Photomicrograph of vehicle control group showed normal fibrillary background. The neuronal cell morphology and tissue architecture was maintained [Figure 1A]. Photomicrograph of pathogenic control group showed coagulative necrosis and vacuolar changes [Figure 1B]. Photomicrographs of aqueous extract of *Embelia ribes* (100 and 200 mg/kg) treated groups showed normal fibrillary background and absence of degenerative changes or necrosis. The neuronal cell morphology and tissue architecture was retained [Figures 1C and 1D]. Photomicrographs of folic acid (100 mg/kg) treated group showed normal fibrillary background, without any pathological symptoms [Figure 1E]. Photomicrographs of the aqueous extract of *Embelia ribes* (100 and 200 mg/kg) *per se* groups showed normal fibrillary background and neuronal cell morphology [Figures 1F and 1G].

## Discussion

Hyperhomocysteinemia has recently emerged as an independent risk factor for the development of coronary, cerebrovascular and peripheral arterial occlusive disease.[17] Elevated homocysteine promotes atherosclerosis through increased oxidative stress, impaired endothelial function and induction of thrombosis. The role of oxidative stress in neurodegeneration has been intensively studied. Oxidative stress was one important mechanism for homocysteine toxicity in neuronal cells.[18] Antioxidant treatment restores several toxic effects of homocysteine.[19]

Bhandari *et al.*[12,13] reported the diabetic dyslipidemic and antioxidant activity of *Embelia ribes* Burm in streptozotocin-induced diabetes in rats, using gliclazide as a positive control drug. In the present study, we examined the homocysteine and lipid lowering potential of the aqueous extract of *Embelia ribes* (100 and 200 mg/kg, p.o.) in methionine-induced hyperhomocysteinemia and hyperlipidemia in rats.

Several potential mechanisms underlying the deleterious effect of homocysteine in the brain have been proposed, which include oxidative stress, alterations in DNA methylation and activation of the excitotoxic NMDA receptors.[5] Hyperhomocysteinemia may promote the generation of reactive oxygen species (ROS) such as H_2_O_2_ and hydroxyl radicals via the auto-oxidation of sulfhydryl (-SH) group[20] or by decreasing the intracellular levels of GSH that are involved in the elimination of free radicals. Homocysteine, a thiol containing amino acid derived from demethylation of dietary methionine, may generate partially reduced ROS that are able to stimulate the lipid peroxidation involved in the atherosclerotic process. Thus, an imbalance in dietary methionine may contribute to the development of atherosclerosis by increasing homocysteine levels.[21]

The data in our present study showed that methionine (1 g/kg, p.o.) treatment for 30 days in pathogenic control group (Group II) rats significantly (*P* < 0.01) elevated the levels of serum homocysteine, LDH, total cholesterol, triglycerides, LDL-C and VLDL-C and atherosclerotic index values. Further, there was a decrease in serum HDL-C levels, with decrease in GSH content and increase in LPO levels in brain homogenates.

Atherogenic index indicates the deposition of foam cells or plaque or fatty infiltration or lipids in heart, coronaries, aorta, liver and kidneys. The higher the atherogenic index, the higher is the risk of the above organs for oxidative damage.[22]

Free radicals generated by hyperhomocysteinemia, initiate lipid peroxidation of the membrane bound polyunsaturated fatty acids, leading to the impairment of the structural and functional integrity of the membrane.[23] This concurs with the present findings, wherein the levels of LPO were found to be significantly (*P* < 0.01) increased in the animals subjected to methionine treatment. Due to this increased lipid peroxidation, GSH levels are lowered.[24]

In the present study, elevated levels of homocysteine, LDH, total cholesterol, triglycerides, LDL-C and VLDL-C in serum and LPO in brain homogenates were reduced significantly (*P* < 0.01) after treatment with aqueous extract of *Embelia ribes*. Further, there was a decrease in the atherogenic index values, suggesting antihyperhomocysteinemic and antihyperlipidemic potential of *Embelia ribes*. Further, the levels of HDL-C in serum and GSH in brain homogenates were increased significantly (*P* < 0.01), thereby, enhancing the endogenous antioxidant levels. And also, the results of test drug were comparable to folic acid, a standard positive control.

Biochemical assay of various parameters in serum and brain tissues of the animals revealed that aqueous extract of *Embelia ribes* in both the doses favorably modified various biochemical markers in methionine-induced hyperhomocysteinemic rats significantly (*P* < 0.01), as compared to pathogenic hyperhomocysteinemic rats.

Chemically, *Embelia ribes* is reported to contain embelin, quercitol (polyphenol), tannins and alkaloids,[25] which may contribute to its antioxidant activity. In the present study, on standardization of the aqueous extract of *Embelia ribes,* it was found to contain alkaloids, carbohydrates, flavonoids, phenolic compounds, proteins and saponins. It can thus be concluded that the antioxidant effect of the aqueous extract of *Embelia ribes* can be due to the content of alkaloids, flavonoids, phenolic compounds and saponins.

Lastly, the light microscopic observations of the brain tissues of the aqueous extract of *Embelia ribes* treated animals exhibited near normal pattern, thereby further supporting its role as a promising neuroprotective agent.

## Conclusion

The present results provide clear evidence that aqueous extract of *Embelia ribes* treatment enhances the antioxidant defense against methionine-induced hyperhomocysteinemia, hyperlipidemia and oxidative stress in brain. On the basis of above findings, it can be assumed that aqueous extract of *Embelia ribes* could be a potential source for a novel discovery for central nervous system disorders.
